# A decade of cardiac surgery after transcatheter aortic valve replacement: Short-term clinical outcomes at a high-volume center

**DOI:** 10.1016/j.xjse.2026.100128

**Published:** 2026-05-04

**Authors:** Marco Tagliafierro, Ali Fatehi Hassanabad, Jonathan Nickles, Riley Sevensky, Arnar Geirsson, Isaac George, Hiroo Takayama, Michael Argenziano, Luigi Pirelli

**Affiliations:** Cardiothoracic Surgery Department, Columbia University Irving Medical Center, New York, NY

**Keywords:** TAVR explant, Redo surgery, TAVR dehiscence, valve reoperation, TAVR complications

## Abstract

**Background:**

The growing adoption of transcatheter aortic valve replacement (TAVR) in younger and lower-risk patients has increased the number of patients with other cardiac diseases requiring surgical intervention. Such surgeries pose unique technical challenges due to the presence of the TAVR, often requiring explantation. Despite increasing clinical relevance, outcomes of these operations remain poorly characterized. We sought to assess the incidence, indications, and short-term results of cardiac surgery following TAVR at a high-volume institution.

**Methods:**

This was a retrospective single-center analysis of patients undergoing any cardiac surgery post-TAVR between 2015 and 2024. Primary endpoints were perioperative all-cause mortality and stroke; secondary endpoints included cardiopulmonary bypass and cross-clamp times, as well as in-hospital and 30-day outcomes.

**Results:**

Among 10,898 surgeries, 61 (0.5%) involved patients with prior TAVR (median age, 72 years; 59% male), including 85% with hypertension, 28% with diabetes, 43% with chronic lung disease, and 15% with cerebrovascular disease. The median time between TAVR and surgery was 20 months, and 57% of the surgeries were urgent or emergent/salvage procedures. Major indications included TAVR dysfunction (28%), infective endocarditis (26%), and aortic pathology (13%). Common procedures were TAVR explant and surgical aortic valve replacement (n = 49), mitral surgery (n = 19), aortic root/arch surgery (n = 12), and multivessel coronary artery bypass grafting (n = 5). The median aortic cross-clamp and cardiopulmonary bypass times were 121 minutes and 160 minutes, respectively. The mortality rate was 13%. Other outcomes included stroke (3%), prolonged ventilation (31%), tracheostomy (7%), de novo dialysis (8%), need for postoperative blood products (61%), cardiac reintervention (10%), discharge to rehabilitation facility (34%), and readmission (13%).

**Conclusions:**

Cardiac surgery post-TAVR is uncommon and associated with significant morbidity and mortality. Prosthesis dysfunction and endocarditis are the leading indications, and TAVR explant remains a common although highly morbid intervention.

## Graphical Abstract

**Figure:**
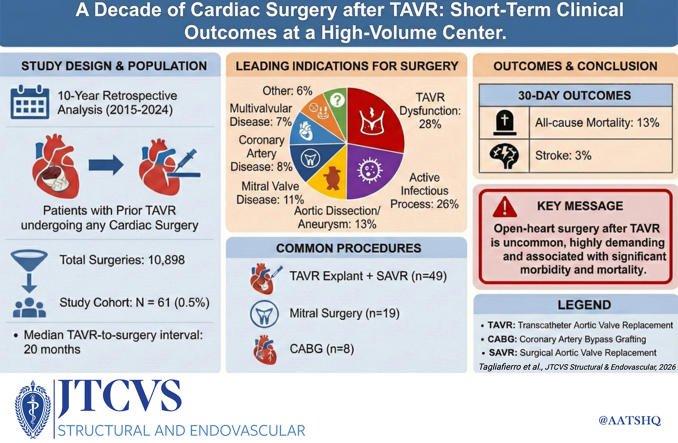

**Figure:**
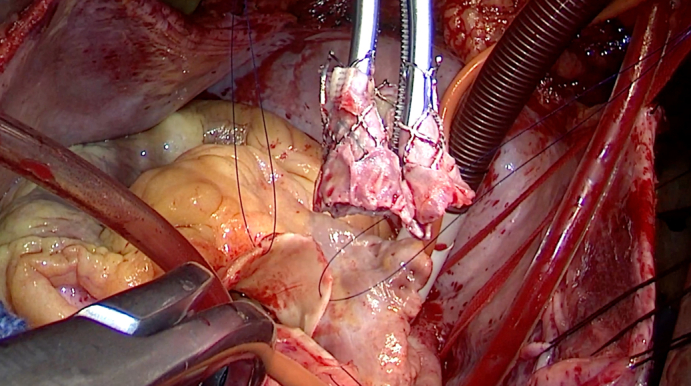
Intraoperative view of a TAVR explant.

**Central Message**

Open-heart surgery after TAVR is an uncommon, highly demanding operation the carries significant morbidity and mortality. This study highlights the importance of careful planning and risk stratification.

**Perspective**

Given the longer life expectancy and the widespread use of transcatheter interventions even in younger and healthier individuals, TAVR explant is currently the fastest growing cardiac surgical procedure in the United States. These procedures carry intrinsic risks and significant morbidity and mortality, owing to the technical aspects of the surgery and to the distinct risk profile of this patient population.

Transcatheter aortic valve replacement (TAVR) continues to play an important role in the lifetime management of aortic valve disease. It is now an established alternative to surgical aortic valve replacement (SAVR) across all patient risk profiles. Following initial approval for inoperable patients,^1^^,^^2^ contemporary studies have demonstrated TAVR to be noninferior to SAVR even in intermediate-risk and low-risk patients.^3^, ^4^, ^5^, ^6^ As a result, the number of patients undergoing TAVR has increased exponentially in recent years, with the procedure now surpassing SAVR as the predominant treatment modality for aortic valve replacement.^7^

Given the rapidly expanding use of TAVR in younger and lower-surgical-risk populations, there is an emerging subset of patients with previously implanted TAVR valves who require subsequent cardiac surgery.^8^ The Society of Thoracic Surgeons (STS) recently reported that TAVR explant is currently the fastest growing cardiac surgical procedure in the United States, with a 150% annual growth rate.^9^ Such operations may be indicated for a variety of reasons, including prosthetic valve deterioration or malposition, prosthetic valve infective endocarditis (IE), progression of nonaortic valvular disease, coronary artery disease, and pathologies of the aortic root and arch.^10^, ^11^, ^12^

These patients represent a new clinical challenge, as cardiac operations performed in the presence of a TAVR valve entail unique technical complexities. Consequently, the active involvement of cardiovascular surgeons on the multidisciplinary Heart Team is paramount during the initial evaluation for TAVR. Particularly in younger and lower-risk patients, the team must carefully weigh the immediate procedural strategy advantage against the lifetime management of the patient's structural heart disease, considering the potential significant morbidity and mortality of future surgical interventions, especially when a TAVR explant is required (Video 1).

Despite a rapid rise in the need for cardiac surgery after TAVR,^8^ the relevant literature consists primarily of registry analyses focused on TAVR explantations.^13^, ^14^, ^15^ The present study aimed to characterize the incidence, indications, and outcomes of all cardiac surgical procedures in patients with a previous TAVR at a high-volume academic center over 10 years.

## Methods

### Patient Population and Data Collection

Following Institutional Review Board approval (IRB-AAAV5722; latest date of approval January 30, 2026; informed consent waived due to the study’s retrospective nature), data were collected retrospectively from all patients presenting at Columbia University Medical Center with a history of prior TAVR who underwent any cardiac surgery between 2015 and 2024. Data relevant to the study were collected retrospectively from the institutional STS/American College of Cardiology Transcatheter Valve Therapy Registry and the STS Adult Cardiac Surgery Database, supplemented with chart review as necessary. Owing to the unavailability of the STS SAVR post-TAVR risk assessment calculator at the time of data analysis, and because all other available calculators do not allow inclusion of TAVR explant as part of the operation (thus significantly underestimating the surgical risk), preoperative STS Predicted Risk of Mortality and morbidity scores could not be computed for this cohort. Consequently, observed-to-expected ratios of postoperative outcomes were not estimable.

### Primary and Secondary Endpoints

The primary endpoints were the incidences of in-hospital and 30-day all-cause mortality and stroke. Secondary endpoints included aortic cross-clamp and cardiopulmonary bypass times, intensive care unit (ICU) length of stay, postoperative blood product use, renal status (ie, creatinine level and new-onset renal replacement therapy), postoperative ventilation time, prolonged ventilation (>24 hours), tracheostomy rates, cardiac reinterventions, discharge location, and 30-day in-hospital readmissions for any cause. Subgroup analyses for all endpoints were performed comparing patients by surgical status and by procedure type (ie, isolated procedure vs multiple procedures), and also comparing patients who underwent TAVR explant and those whose transcatheter heart valve (THV) was left in situ.

### Statistical Analysis

All statistical analyses were conducted using R version 4.4.2. Continuous variables were assessed for normality with the Shapiro-Wilk test. Normally distributed data are presented as mean ± standard deviation. Variables with non-normal distributions are reported as median (interquartile range [IQR]). Categorical variables are summarized as count and percentage. Group comparisons for continuous variables were performed using the Wilcoxon rank-sum test, and categorical variables were compared using the χ^2^ test or Fisher exact test, as appropriate. A *P* value <.05 was considered statistically significant.

## Results

### Baseline Patient Characteristics

Table 1 summarizes the baseline patient demographics. Sixty-one patients with a history of TAVR underwent cardiac surgery, including 36 males (59%). The patients’ mean age was 72 years, and their mean body mass index was 28.5 kg/m^2^. Hypertension was present in 52 patients (85%), and 9 (15%) had a history of stroke, 3 [33%] of whom in the 30 days prior to the operation). Preoperatively, 25 patients (41%) presented with symptoms consistent with New York Heart Association class III or IV, along with a median left ventricular ejection fraction of 57% (IQR, 55%-63%). The median time from TAVR to a cardiac operation was 20 (IQR, 7-50) months.

**Table 1:** Baseline patient characteristics

Variable	TAVR explant (N = 49)	No TAVR explant (N = 12)	Overall (N = 61)	*P* value
Age, y, mean ± SD	72.2 ± 9.1	73.5 (9.0)	72.4 (9.0)	.643
Male sex, n (%)	29 (59)	7 (58)	36 (59)	1.000
BMI, kg/m^2^, mean ± SD	28.4 ± 5.6	28.8 ± 6.0	28.5 ± 5.7	.765
Cardiovascular risk factors, n (%)				
Hypertension	42 (86)	10 (83)	52 (85)	1.000
Diabetes mellitus	13 (27)	4 (33)	17 (28)	.723
Obstructive sleep apnea	7 (14)	3 (25)	10 (16)	.397
Peripheral artery disease	4 (8)	0 (0)	4 (7)	.576
COPD, n (%)	21 (43)	5 (42)	26 (43)	1.000
Preoperative dialysis, n (%)	2 (4)	0 (0)	2 (3)	1.000
Cerebrovascular disease, n (%)	18 (37)	4 (33)	22 (36)	1.000
CVA, n (%)	9 (18)	1 (8)	10 (16)	.670
≤30 d	3 (33)	0 (0)	3 (30)	1.000
>30 d	6 (67)	1 (100)	7 (70)	1.000
Liver disease, n (%)	1 (2)	0 (0)	1 (2)	1.000
Previous AMI, n (%)	8 (16)	4 (33)	12 (20)	.229
History of mediastinal radiation, n (%)	3 (6)	1 (8)	4 (7)	1.000
Endocarditis, n (%)	17 (35)	1 (8)	18 (30)	.089
Atrial fibrillation, n (%)	21 (43)	7 (58)	28 (46)	.356
LVEF, %, median (IQR)	58 (55-63)	52 (40-58)	57 (55-63)	.056
Reduced (<50%), n (%)	8 (16)	5 (42)	13 (21)	.108
NYHA class >II, n (%)	19 (39)	6 (50)	25 (41)	.736
Previous cardiac surgeries, n (%)				
TAVR, n (%)	49 (100)	12 (100)	61 (100)	—
Time since TAVR, mo, median (IQR)	15.6 (6.9-46.9)	18.8 (10.8-35.1)	17.2 (6.9-46.9)	.977
PCI, n (%)	14 (29%)	4 (33)	18 (30)	.736
CABG, n (%)	5 (10%)	3 (25)	8 (13)	.183

*TAVR*, Transcatheter aortic valve replacement; *BMI*, body mass index; *COPD*, chronic obstructive pulmonary disease; *CVA*, cerebrovascular accident; *AMI*, acute myocardial infarction; *LVEF*, left ventricular ejection fraction; *IQR*, interquartile range; *NYHA*, New York Heart Association; *PCI*, percutaneous coronary intervention; *CABG*, coronary artery bypass grafting.

The most common indication for surgery was noninfectious TAVR dysfunction (n = 17; 35%). Other frequent indications included IE (n = 16 [26%], with 14 [88%] affecting the THV and 2 [12%] affecting the mitral valve [MV]), aortic disease (n = 8; 13%), de novo MV disease (n = 7; 11%), progressive multivessel coronary disease (n = 5; 8%), multivalvular (ie, THV and MV, with or without the tricuspid valve) disease (n = 4; 7%]), and orthotopic heart transplantation (n = 2 [3%]). Comparing patients undergoing TAVR explant with those whose THV was left in situ revealed no significant baseline differences.

### Intraoperative Variables

Table 2 summarizes the intraoperative data. Less than one-half of the procedures were elective (43%), 9% were emergent or salvage, and the remaining 49% were urgent. Isolated procedures were a minority, corresponding to 20 cases (33%) cases, while all other 41 surgeries had multiple cardiac interventions. TAVR explant and subsequent SAVR were performed in 49 patients (80%); MV interventions, in 19 patients (31%; including 1 repair with annuloplasty [5%] and 18 replacements [95%]); coronary artery bypass grafting, in 8 (13%;, including 3 [38%] single vessel, 2 [25%] double vessel, 2 [25%] triple vessel, and 1 [13%] quadruple vessel; and tricuspid valve interventions in 9 (15%, including 8 repairs with annuloplasty (89%) and 1 replacement (11%). Intraoperative blood products were used in 36 patients (59%). The volume of operations per surgeon varied, with the 3 most experienced surgeons performing 21 (34%), 11 (18%), and 10 (16%) of the cases.

**Table 2:** Intraoperative data

Variable	Value
Admission status, n (%)	
Elective	26 (43)
Urgent	30 (49)
Emergent	4 (7)
Salvage	1 (2)
Primary indication for surgery, n (%)	
Noninfectious TAVR dysfunction	17 (28)
Severe AS	9 (53)
Moderate PVL	4 (24)
Valve-in-valve impossible due to risk of PPM	3 (18)
Coronary occlusion secondary to TAVR deployment	1 (6)
IE	16 (26)
IE on THV	14 (88)
IE on MV	2 (12)
Aortic dissection/aneurysm/pseudoaneurysm	8 (13)
Mitral valve disease	7 (11)
Multivessel coronary disease	5 (8)
Multivalvular disease	4 (7)
Orthotopic heart transplant	2 (3)
Other	2 (3)
Isolated procedures, n (%)	20 (33)
Operations, n (%)	
TAVR explant + AVR	49 (80)
Mitral valve intervention	19 (31)
Mitral valve repair (annuloplasty alone)	1 (5)
Mitral valve replacement	18 (95)
Aortic root/arch surgery	12 (20)
Tricuspid valve intervention	9 (15)
Tricuspid valve repair (annuloplasty alone)	8 (89)
Tricuspid valve replacement	1 (11)
CABG	8 (13)
1 vessel	3 (38)
2 vessels	2 (25)
3 vessels	2 (25)
4 vessels	1 (13)
Orthotopic heart transplant	2 (3)
ECMO	6 (10)
Left atrial appendage ligation	10 (16)
ASD/PFO closure	6 (10)
Other	8 (13)
Intraoperative blood products use, n (%)	36 (59)

*TAVR*, Transcatheter aortic valve replacement; *AS*, aortic stenosis; *PVL*, paravalvular leak; *PPM*, patient–prosthesis mismatch; *IE*, infective endocarditis; *THV*, transcatheter heart valve; *MV*, mitral valve; *AVR*, aortic valve replacement; *CABG*, coronary artery bypass grafting; *ECMO*, extracorporeal membrane oxygenation; *ASD*, atrial septal defect; *PFO*, patent foramen ovale.

### Primary and Secondary Endpoints

The study’s primary and secondary endpoints are listed in Table 3, Table 4, Table 5. No patient died intraoperatively, but 8 (13%) died within 30 days postsurgery; more specific information on the preoperative characteristics of these patients is provided in Online Data Supplement. Of these 8 patients, 6 had a TAVR explant. One patient developed cardiogenic shock secondary to right ventricular dysfunction. Another patient died after a prolonged ICU course complicated by renal failure requiring hemodialysis, complete heart block, and toxic metabolic encephalopathy. A third patient died after experiencing tension pneumothorax, worsening right ventricular dysfunction, and cardiogenic shock necessitating peripheral venoarterial extracorporeal mechanical oxygenation, and septic shock. The fourth patient died after liver and kidney failure requiring dialysis and septic shock. The fifth patient died after severe right ventricular dysfunction with cardiogenic and profound vasodilatory shock necessitating hemodynamic support and venoatrial extracorporeal membrane oxygenation, followed by embolic stroke and ischemic leg ischemia, thrombocytopenia, and gastrointestinal bleeding. The sixth patient presented for an elective TAVR complicated by aortic dissection extending to the descending aorta and involving both carotid arteries, requiring an extensive but unsuccessful intervention. Two deaths occurred in patients who underwent MV replacement without THV explant: one because of cardiogenic shock and respiratory failure with prolonged intubation and renal failure requiring hemodialysis and the other from multiorgan failure after combined septic and cardiogenic shock, prolonged ventilation followed by tracheostomy for respiratory failure, and hemodialysis for renal failure.

**Table 3:** Postoperative data stratified by surgical status

Variable	Elective or urgent (N = 56)	Emergent or salvage (N = 5)	Overall (N = 61)	*P* value
Primary endpoints, n (%)				
Mortality	7 (12)	1 (20)	8 (13)	.518
Cerebrovascular accident	2 (4)	0 (0)	2 (3)	1.000
Secondary endpoints				
CPB time, min, median (IQR)	160 (102-217)	203 (140-286)	160 (106-218)	.314
Aortic cross-clamp time, min, median (IQR)	122 (80-146)	158 (88-224)	122 (80-150)	.587
ICU total length of stay, h, median (IQR)	113.1 (51.9-186.9)	150.0 (141.0-276.5)	113.8 (52.9-203.0)	.275
Postoperative renal status				
Postoperative renal failure requiring new HD, n (%)	5 (9)	0 (0)	5 (8)	1.000
Postoperative creatinine, mmol/L, median (IQR)	1.2 (0.9-1.6)	1.3 (1.2-1.5)	1.2 (0.9-1.6)	.510
Ventilation, h, median (IQR)	13.1 (6.7-26.3)	39.7 (17.8-138.1)	13.6 (7.4-35.1)	.080
Prolonged ventilation (>24 h), n (%)	16 (29)	3 (60)	19 (31)	.170
Reintervention due to cardiac causes, n (%)	6 (11)	0 (0)	6 (10)	1.000
Permanent pacemaker placement, n (%)	5 (83)	0 (0)	5 (83)	—
Implantable cardiac defibrillator, n (%)	1 (17)	0 (0)	1 (17)	—
Rehospitalizations, n (%)	8 (14)	0 (0)	8 (13)	1.000

*CPB*, Cardiopulmonary bypass; *IQR*, interquartile range; *ICU*, intensive care unit; *HD*, hemodialysis.

**Table 4:** Postoperative data stratified by procedure type

Variable	Isolated (N = 20)	Concomitant (N = 41)	Overall (N = 61)	*P* value
Primary endpoints, n (%)				
Mortality	1 (5)	7 (17)	8 (13)	.253
Cerebrovascular accident	0 (0)	2 (5)	2 (3)	1.000
Secondary endpoints				
CPB time, min, median (IQR)	92 (69-147)	181 (139-226)	160 (106-218)	<.001
Aortic cross-clamp time, min, median (IQR)	69 (54-108)	125 (100-162)	122 (80-150)	<.001
ICU total length of stay, h, median (IQR)	58.8 (34.7-133.2)	127.0 (91.2-276.5)	113.8 (52.9-203.0)	.012
Postoperative renal status				
Postoperative renal failure requiring new HD, n (%)	1 (5)	4 (10)	5 (8)	1.000
Postoperative creatinine, mmol/L, median (IQR)	0.9 (0.8-1.4)	1.2 (1.0-1.8)	1.2 (0.9-1.6)	.082
Ventilation, h median (IQR)	8.9 (5.2-17.7)	17.8 (9.6-90.9)	13.6 (7.4-35.1)	.097
Prolonged ventilation (>24 h), n (%)	3 (15)	16 (39)	19 (31)	.079
Reintervention due to cardiac causes, n (%)	2 (10)	4 (10)	6 (10)	1.000
Permanent pacemaker placement, n (%)	1 (50)	4 (100)	5 (83)	—
Implantable cardiac defibrillator, n (%)	1 (50)	0 (0)	1 (17)	—
Rehospitalizations, n (%)	1 (5)	7 (17)	8 (13)	.253

*CPB*, Cardiopulmonary resuscitation; *IQR*, interquartile range; *ICU*, interquartile range; *HD*, hemodialysis.

**Table 5:** Postoperative data stratified by TAVR explant status

Variable	TAVR explant (N = 49)	No explant (N = 12)	Overall (N = 61)	*P* value
Primary endpoints, n (%)				
Mortality	6 (12)	2 (8)	8 (13)	1.000
Cerebrovascular accident	1 (2)	1 (8)	2 (3)	.357
Secondary endpoints, median (IQR)				
CPB time, min	173 (118- 224)	133 (86-152)	160 (106-218)	.028
Aortic cross-clamp time, min	125 (90-159)	86 (63-100)	122 (80-150)	.025
ICU total length of stay, h	113.8 (52.9-181.6)	108.5 (60.8-277.9)	113.8 (52.9-203.0)	.792
Postoperative renal status				
Postoperative renal failure requiring new HD, n (%)	3 (6)	2 (17)	5 (8)	.252
Postoperative creatinine, mmol/L, median (IQR)	1.2 (0.9-1.6)	1.1 (0.9-1.5)	1.2 (0.9-1.6)	.863
Ventilation, h, median (IQR)	13.3 (6.2-28.4)	15.0 (7.9-57.9)	13.6 (7.4-35.1)	.580
Prolonged ventilation (>24 h), n (%)	14 (29)	5 (42)	19 (31)	.489
Reintervention due to cardiac causes, n (%)	5 (10)	1 (8)	6 (10)	1.000
Permanent pacemaker placement, n (%)	5 (100)	0 (0)	5 (83)	—
Implantable cardiac defibrillator, n (%)	0 (0)	1 (100)	1 (17)	—
Rehospitalization, n (%)	5 (10)	3 (25)	8 (13)	.183

A permanent cerebrovascular event occurred in 2 patients (3%). The median cardiopulmonary bypass and aortic cross-clamp times were 160 (IQR, 106-218) minutes and 122 (IQR, 80-150) minutes, respectively. Five patients (8%) required de novo hemodialysis, and 37 (61%) required blood products postoperatively. The median duration of invasive ventilatory support was 13.6 (IQR, 7.4-35.1) hours; 19 patients (31%) remained on the ventilator for >24 hours, and 4 (7%) required a tracheostomy. Six patients (10%) required a reintervention for cardiac causes, including 5 for implantation of a permanent pacemaker and 1 for implantation of an implantable cardioverter-defibrillator. Twenty-one patients (34%) were discharged to a rehabilitation facility. Eight patients (13%) were readmitted within 30 days following discharge, 5 for cardiovascular causes, including heart failure exacerbation.

Comparing the subgroups revealed no significant differences in outcomes based on surgical status. However, ICU length of stay (*P* = .012) was significantly longer in cases including a TAVR explant. Similarly, intraoperative times were significantly longer whenever a TAVR was explanted, as well as whenever multiple surgeries were performed. No differences in clinical outcomes were observed in any of the subgroup analyses.

## Discussion

As the indications for TAVR continue to expand, it is likely that a growing number of patients with a THV will require cardiac interventions. Nonetheless, contemporary literature has yet to fully elucidate the outcomes of patients with a history of TAVR who subsequently undergo cardiac surgery. Current evidence in this area is sparse and limited to studies with relatively small sample sizes and short follow-up periods.^8^^,^^14^^,^^16^, ^17^, ^18^, ^19^, ^20^, ^21^ The fact that our study identified only 61 cases, representing just 0.5% of the 10,898 total cardiac surgeries performed over a decade at our quaternary center, underscores the true rarity of these procedures. Given that TAVR explant is currently the fastest-growing cardiac surgery in the United States, sharing real-world experiences from high-volume centers is essential to bridge this knowledge gap. Therefore, ongoing work is required to better understand the outcomes of post-TAVR cardiac surgery.

**Figure 1:**
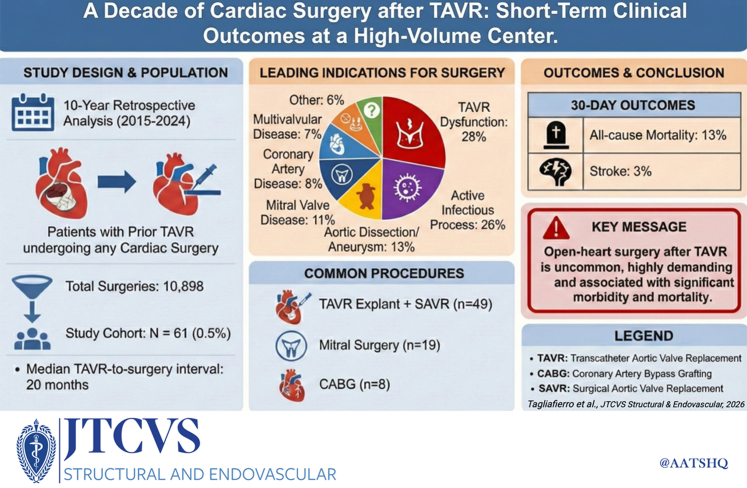
Graphical abstract of the study.

This retrospective single-center analysis presents the clinical outcomes of patients with a history of TAVR undergoing cardiac surgery at our institution over 10 years. Several key observations emerge from our study: (1) cardiac surgery after TAVR remains rare, accounting for only a small fraction of all cardiac operations in a high-volume center; (2) valve pathologies, particularly TAVR dysfunction and THV IE, were the leading indications for surgery; and (3) cardiac surgery after TAVR was associated with considerable morbidity and mortality, reflecting the high-risk profile and procedural complexity of this patient population. The main findings are summarized in Figure 1.

Reflecting a broader trend, patients in our series are similar to those reported in other single-center studies.^17^^,^^18^^,^^20^ Given our evolving understanding of THV durability, examining the profiles of the patients in our cohort is important. Eighty percent of the patients in our series underwent THV explantation and subsequent SAVR. The median interval from TAVR to a subsequent cardiac operation was 20 months, and 17 patients (28%) underwent surgery to address a noninfectious dysfunctional THV. Of the latter operations, 9 (53%) were due to severe aortic stenosis of the THV, 4 (24%) were due to moderate paravalvular leak, 3 (18%) because a valve-in-valve TAVR would have led to patient–prosthesis mismatch, and 1 (6%) because of a coronary occlusion after TAVR. Of note, in their series, Yun and colleagues^18^ found that the interval from TAVR to cardiac surgery decreased from 7 years to <1 year over a 10-year period. While IE represents unpredictable late complications, the high rate of early TAVR failure underscores the need for refined patient selection as TAVR expands to lower-risk cohorts. Owing to the high morbidity and mortality risk of cardiac operations post-TAVR, a more thorough evaluation of patients undergoing elective AVR should be performed whenever other cardiac diseases (eg, nonocclusive coronary obstruction, mild nonaortic valvular disease) are present, as these may progress in the future.

Presently, cardiac surgery after TAVR carries significant morbidity and mortality for a multitude of reasons. Most patients with a THV underwent TAVR at a time when it was reserved for high-risk patients, and thus they are likely to have significant comorbidities. Moreover, operations involving THV explantation are complex secondary to suboptimal exposure and need for more extensive root reconstruction. Indeed, according to the STS Adult Cardiac Surgery Database, among patients requiring TAVR explantation, 30-day mortality is 13.9%, and for those requiring concomitant procedures, most commonly aortic repair, 30-day mortality approaches 20%.^10^ Similarly, the EXPLANT-TAVR International Registry reported 76.1% overall survival at last follow-up, whereas in-hospital, 30-day, and 1-year mortality rates were 11.9%, 13.1%, and 28.5%, respectively.^22^ Outcomes are similarly concerning for patients requiring non–aortic valve cardiac surgery post-TAVR. In a recent analysis of the STS Adult Cardiac Surgery Database, patients who underwent non–aortic valve cardiac operations after TAVR between 2011 and 2019 had a 30-day mortality of 17%.^19^ In our series, over a 10-year period, despite an elevated surgical risk profile, only 8 patients (13%) died and 2 (3%) experienced a cerebrovascular event. These findings closely mirror those reported from the STS Adult Cardiac Surgery Database, in which recent mortality data range between 9% and 17% for patients with a history of TAVR undergoing subsequent cardiac surgery.^8^^,^^14^^,^^17^^,^^21^

Given the low numbers, it will be difficult to ascertain whether the type of surgery post-TAVR can affect outcomes in the present series. Nonetheless, it is important to note that TAVR explant and subsequent SAVR was performed in 80% of patients, MV interventions in 31%, CABG in 13%, and tricuspid valve interventions in 15%. In the present series, in patients who required TAVR explant, the duration and complexity of the procedure, in addition to comorbidities, appeared to have contributed to poor outcomes. With larger sample sizes, ongoing work should explore whether the type of operation post-TAVR can impact outcomes.

In our cohort, more than one-half of the cases involved explantation of the THV. In our experience, the presence of a THV affects both subsequent aortic valve and nonvalvular cardiac surgery, adding an extra layer of complexity in the preoperative and intraoperative phases. In the preoperative stage, multimodal imaging plays a critical role in surgical planning. Coronary anatomy and the location of coronary ostia with respect to the THV can inform cardioprotective strategies. Preoperative imaging, including computed tomography and transesophageal echocardiography, is important to determine whether aortic root surgery is needed or should be anticipated. Similarly, in those who need proximal thoracic aortic surgery, the presence and the type of THV (eg, short vs tall frame) can determine the appropriate location of the aortotomy and where the distal aortic anastomosis can be performed with a synthetic tube graft. We have found that in patients who require post-TAVR MV surgery, the implanted THV can compromise surgical exposure of the MV and also impact the aortomitral curtain. While in some of these cases, the THV can be left intact and still have appropriate exposure to the mitral annulus, other cases require removal of the TAVR valve to appropriately identify the anterior annulus and the trigones. Therefore, the presence of a THV influences both the type of operation and the surgical approach (eg, sternotomy vs thoracotomy vs robot-assisted).

It is reasonable to expect associations among the presence of a THV, the complexity of the indicated subsequent operation, and clinical outcomes. It also is clinically relevant and critical to determine the presence and impact of a learning curve that may be present in this patient population. It is reasonable to expect that surgeon and center volumes will have an impact on outcomes. While our analysis did not reveal any temporal or surgeon volume-dependent associations with respect to outcomes, future studies with large sample sizes and long-term outcomes should closely follow these metrics. Indeed, a learning curve will have to be navigated for these patients and higher-volume centers that have a long history of performing cardiac surgery post-TAVR, especially if a TAVR explant is needed, may portend better outcomes.

Furthermore, a multidisciplinary Heart Team approach and appropriate patient selection are essential to optimize the lifetime management of aortic valve disease. Such decision making should balance the purported benefits of TAVR and the risks associated with potential subsequent valvular and nonvalvular cardiac operation. Finally, while our study offers real-world insights from a high-volume center, future work should characterize TAVR failure modes according to native aortic valve anatomy and disease etiology (eg, bicuspid valves, calcification). Given that early TAVR structural valve degeneration and reintervention are highly morbid and portend overall dismal outcomes, it is important to refine patient selection, identify the patients at risk for future cardiac interventions, and offer the most comprehensive treatment approach that provides the more durable results over time.

### Limitations

This study is intrinsically limited by its single-center, retrospective design. Additionally, selection bias is inherent. Our series includes only Heart Team–selected patients deemed operable post-TAVR, while patients with surgical indications declined for high risk (eg, frailty, comorbidities) were not captured, likely leading to underestimation of overall population mortality. Baseline aortic valve or TAVR implant characteristics (eg, STS Predicted Risk of Mortality score, valve model/size) were unavailable for most patients, because the majority underwent their index TAVR at external institutions, with subsequent referral to our quaternary center for reoperation. Our study focused explicitly on short-term outcomes; while the cohort represents a 10-year surgical experience (2015-2024), we lack the granular, long-term follow-up data necessary to report outcomes beyond the 30-day and in-hospital periods. Furthermore, detailed data on preoperative and postoperative hemodynamic measurements were not uniformly available, hindering a more comprehensive assessment of how these factors might have influenced operative risk and long-term survival. Additionally, because the STS SAVR post-TAVR risk prediction tool was not yet available during the study period, formal calculation of STS preoperative risk scores and corresponding observed-to-expected outcome ratios could not be performed. Similar to other single-center studies, our study has a relatively small sample size. While designing and executing a randomized controlled trial is unlikely for this growing patient population in the near future, with respect to the lifetime management of aortic valve disease, it is incumbent on care providers to make recommendations to patients based on accumulating data on the full spectrum of post-TAVR outcomes, including scenarios in which a subsequent cardiac operation is indicated.

## Conclusions

Cardiac surgery after TAVR is associated with considerable morbidity and mortality. Valvular pathologies, particularly prosthesis dysfunction and endocarditis, are the leading indications for such operations. These findings underscore the importance of long-term planning at the time of initial valve selection and highlight the need for refined risk stratification in this growing patient population.

### Declaration of Generative AI and AI-Assisted Technologies in the Writing Process

Generative AI was not used to generate the scientific content of this manuscript. AI-based tools were used for grammar correction and editing. All content was reviewed and verified by the authors to ensure accuracy and integrity, and the authors take full responsibility for the content of the publication.

### Data Availability Statement

All relevant data are provided in the manuscript and supplemental files. Further information is available on request to the corresponding author.

## Conflict of Interest Statement

AG: consultant for Edwards Lifescience and Medtronic; advisory board member for enableCV and Intuitive Surgical. IG: grant support from Medtronic, Boston Scientific, Edwards Lifesciences; consultant (honoraria) for Zimmer Biomet, Atricure, Neosurgery, Neptune Medical, AbbVie, Johnson & Johnson, Durvena, Boston Scientific, Edwards Lifesciences, Medtronic, Encompass Medical, Summus Medical, Abbott SJM, BCI, Xeltis, Innocardiac, KIS Medical, Shockwave Medical, Retex Medical, and Mediscient AI; advisory board member for Edwards Surgical, Medtronic Surgical, Medtronic Structural Mitral & Tricuspid, Trisol Medical, Valcare Medical, Durvena, AbbVie, Johnson & Johnson, Foldax Medical, Zimmer Biomet, Neosurgery, Boston Scientific, Summus Medical, BCI, Encompass Medical, HVT Medical, Innocardiac, KIS Medical, Retex Medical, Abbott Global TAVI; Equity: Valcare Medical, Durvena, CardioMech, Vdyne, MitreMedical, BCI, Trisol Medical, HVT Medical, Foldax, KIS Medical, Retex Medical, and Mediscent AI; institutional funding to Columbia University: Edwards Lifesciences, Medtronic, Abbott Vascular, Boston Scientific, and JenaValve. HT: consultant for Artivion and Edwards. LP: consultant (honoraria) for Abbott, Edwards Lifesciences, and Medtronic; grant from American College of Cardiology. All other authors reported no conflicts of interest.

The *Journal* policy requires editors and reviewers to disclose conflicts of interest and to decline handling or reviewing manuscripts for which they may have a conflict of interest. The editors and reviewers of this article have no conflicts of interest.

## Supplementary Data

Video 1 Video of a TAVR explant. Video available at: https://www.jtcvs.org/article/S2950-6050(26)00033-1/fulltext.
