# Supplementary figures and images for: A decade of cardiac surgery after transcatheter aortic valve replacement: Short-term clinical outcomes at a high-volume center
Source: JTCVS Struct Endovasc. 2026 May 4;10:100128. doi: 10.1016/j.xjse.2026.100128 (PMC13244778; doi:10.1016/j.xjse.2026.100128)

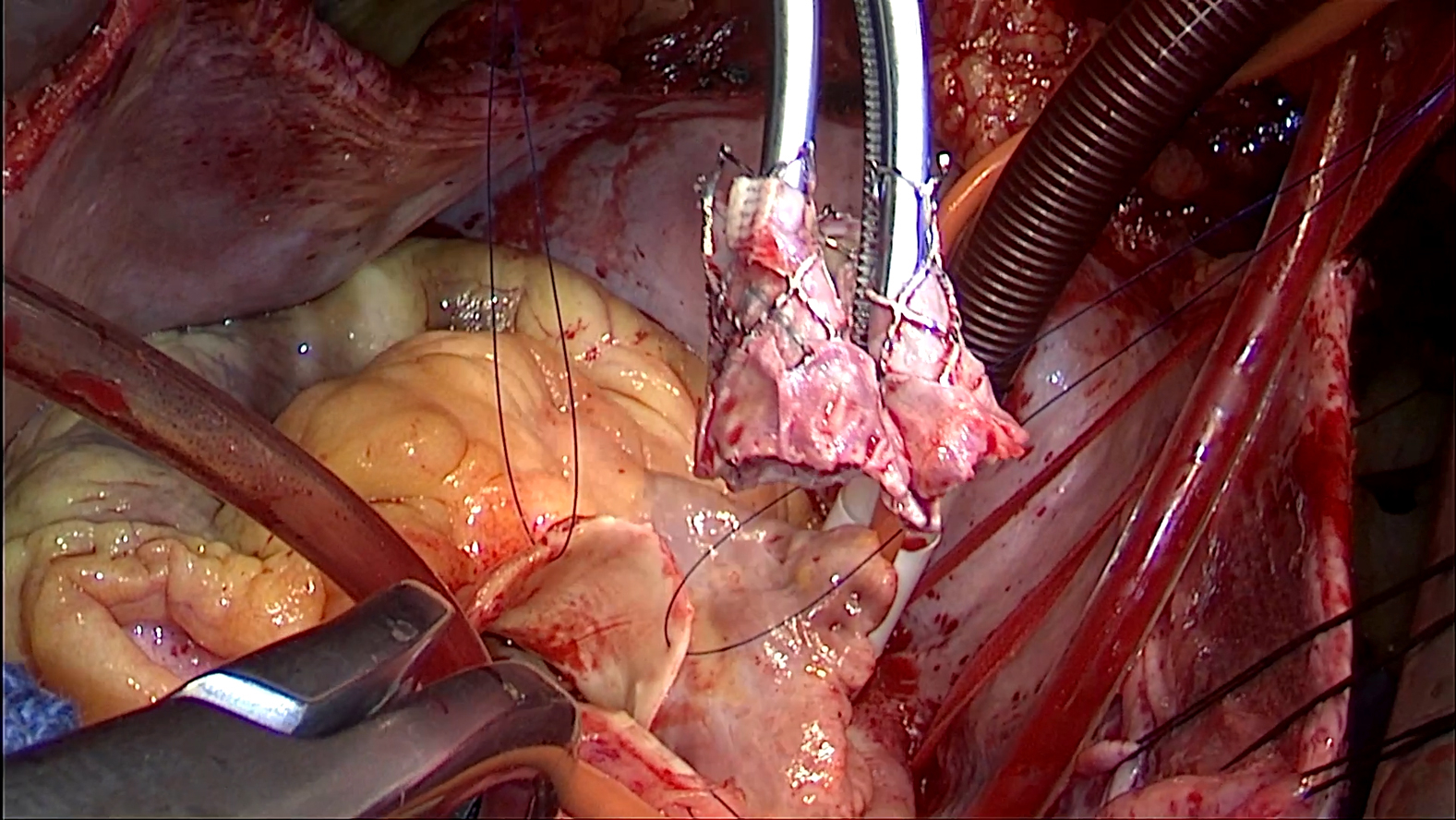

Supplement: Video 1 — Video of a TAVR explant. Video available at: https://www.jtcvs.org/article/S2950-6050(26)00033-1/fulltext. [file fx2.jpg]
