# Supplementary material for: A decade of cardiac surgery after transcatheter aortic valve replacement: Short-term clinical outcomes at a high-volume center
Source: JTCVS Struct Endovasc. 2026 May 4;10:100128. doi: 10.1016/j.xjse.2026.100128 (PMC13244778; doi:10.1016/j.xjse.2026.100128)
Supplement: Online Data Supplement [file mmc2.docx]

**Supplemental Files**

| **Patient number** | **TAVR explant?** | **Operation** | **Surgical status** | **Pre-existing conditions** | **Cause of Death** |
| --- | --- | --- | --- | --- | --- |
| 1 | Yes | TAVR explant + SAVR, MVR, TV annuloplasty | Elective | Hypertension, COPD, CHF, AF | Cardiogenic shock (RV dysfunction) |
| 2 | Yes | TAVR explant + SAVR, MVR | Urgent | Hypertension | Renal failure, complete heart block, toxic metabolic encephalopathy |
| 3 | Yes | TAVR explant + SAVR, MVR, LAAL, ASD closure | Urgent | Hypertension, diabetes mellitus, COPD, CHF | Tension pneumothorax, RV dysfunction, VA ECMO, septic shock |
| 4 | Yes | TAVR explant + SAVR, IABP, ECMO | Urgent | Hypertension, diabetes mellitus, prior MI | Liver/kidney failure, septic shock |
| 5 | Yes | TAVR explant + SAVR, TV replacement | Elective | N/A | RV dysfunction, VA ECMO, embolic strokes, GI bleed |
| 6 | Yes | TAVR explant + SAVR, Type A dissection Repair with Ascending aortic replacement and partial transverse arch repair, Aortic Root Repair, Cabral bypass reconstruction of Left and right coronary artery, ECMO | Salvage | Hypertension, AF | Unsuccessful salvage intervention |
| 7 | No | MV replacement | Elective | Hypertension, diabetes mellitus, OSA, AF | Cardiogenic shock, respiratory/renal failure |
| 8 | No | MV replacement, TV annuloplasty, LAAL | Urgent | Hypertension, CHF, AF | Multiorgan failure, septic/cardiogenic shock |

Supplemental Table 1: Details of postoperative deaths.

| **Time** | **Operative Step** |
| --- | --- |
| 0:03 | Aortotomy with Transverse Incision |
| 0:10 | Left Atrial Appendage Ligation |
| 0:18 | Extension of Aortotomy |
| 0:30 | Initial Inspection of TAVR Valve |
| 0:47 | TAVR Explant with Double Kocher Technique |
| 1:00 | Separation of the Stent Frame from the Aortic Wall |
| 1:51 | Valve Liberation |
| 2:14 | Resection of Leaflet 1 |
| 2:21 | Resection of Leaflet 2 |
| 2:23 | Resection of Leaflet 3 |
| 2:49 | Annular Debridement |
| 3:04 | Final Inspection of Aortic Annulus |

Supplemental Table 2: TAVR Explant with leaflet resection timestamp.
